# Adverse Childhood Experiences are associated with choice of partner, both partners' relationship and psychosocial health as reported one year after birth of a common child. A cross-sectional study

**DOI:** 10.1371/journal.pone.0244696

**Published:** 2021-01-20

**Authors:** Sven-Olof Andersson, Eva-Maria Annerbäck, Hans Peter Söndergaard, Johan Hallqvist, Per Kristiansson

**Affiliations:** 1 Department of Public Health and Caring Sciences, Uppsala University, Uppsala, Sweden; 2 Centre for Clinical Research in Sormland, Uppsala University, Eskilstuna, Sweden; 3 Department of Clinical Neuroscience, Karolinska Institute, Stockholm, Sweden; Toyama Daigaku, JAPAN

## Abstract

Adverse Childhood Experiences (ACEs) are common and known to have consequences for individuals’ adult health, leading to a higher risk of illness. The aims of the study were to investigate the ACEs in couples, to examine the extent of assortative mating and to investigate the association between the relationship of the load of ACEs within couples and health outcomes, one year after the birth of a common child. At antenatal clinics in Sweden 818 couples were recruited and investigated one year after the birth of a common child answering a questionnaire including the exposure to ten ACE categories and several outcome variables. In total, 59% of both mothers and partners reported exposure to at least one of the ten ACE categories. Among the mothers 11% and among the partners 9% reported exposure to ≥4 ACE categories (p = 0.12). There was a correlation between the numbers of ACE categories reported by the mothers and their partners (Spearman’s ρ = 0.18, p<0.001). This association pertained to six of the ten ACE categories. In multiple logistic regression analyses, there were associations between the ACE exposure load and unfavourable outcomes among the mothers, the partners and within the couples. Unfavourable outcomes concerning health were most prominent in couples where both members reported exposures to ≥4 ACE categories (self-rated bad health (OR 13.82; CI 2.75–69.49), anxiety (OR 91.97; CI 13.38–632.07), depression (OR 17.42; CI 2.14–141.78) and perceived stress (OR 11.04; CI 2.79–43.73)). Mothers exposed to ACEs tend to have partners also exposed to ACEs. Exposure to ACEs was associated with bad health and unfavourable life conditions within the couples, especially among couples where both members reported exposure to multiple ACEs. These results should stimulate incentives to find, to support and to treat individuals and couples where both members report multiple ACEs. The consequences for the children should be further studied as well as how these families should be treated in health care and society.

## Introduction

It is well known that the conditions from the beginning of life as well as conditions during childhood form the basis for lifelong health and well-being through positive experiences that strengthen developing biological systems [1, 2]. There is also growing evidence for the effect of early life stress that includes disruption of developing brain architecture, other maturing organs and metabolic functions [3] that have negative consequences for health, morbidity and mortality later in life [4] in absence of protective factors and resilience [2]. Adverse childhood experiences (ACEs) are examples of conditions and circumstances of importance for toxic early life stress that deeply affect children, forming the basis for intergenerational transmission of trauma [5] as well as life and health as adults [4].

At the end of the 20th Century the health maintenance organisation Kaiser Permanente formulated the concept of ACEs. In a study of more than 17000 health maintenance organization members they found a strong graded relationship between exposure to abuse, neglect and family dysfunctions during childhood or adolescence (0–18 years) and multiple risk factors for several diseases and leading causes of death in adults [6]. Since then, there has been a global body of epidemiologic research that has confirmed their results [4, 7–10].

Studies are increasingly establishing how childhood exposure to chronic stress leads to changes in nervous, endocrine and immune systems by inducing physiological abnormalities across allostatic systems, biological aging and health [3, 11]. Chronic stress might also be of importance for the intergenerational transmission of ACEs [5, 12, 13]. Among individuals, the occurrence of one ACE increases the prevalence of exposure to additional ACEs, rather than occurring independently [14]. Individuals with increasing numbers of ACEs show a higher prevalence of many health impairments including some of the leading causes of the global burden of disease [2–4]. On an individual level a graded relationship of the load of ACE exposure to several different outcomes was found by Anda et al. [15].

Marital resemblance (assortative mating) refers to the observed tendency for mated pairs to be more phenotypically similar for a given characteristic than would be expected by chance [16]. Since assortative mating adds to both the genetic and familial environmental risk for illness, it could increase the transmission and persistence of risk factors and specific illnesses in the population, such as cigarette smoking, anxiety, depressive symptoms, major psychiatric diagnoses, diabetes and cancer [16–18]. There is a paucity of studies that focus on the association between marital resemblance and association of multiple ACEs within a family. We have only found one study on ACE and health within couples with economic disadvantage and a racial or ethnic minority background [19]. This study showed a significant pathway between ACEs and health through relationship quality to which the dyadic influence contributed.

To become a parent might be an overwhelming experience that have important physiologic effects on the individuals. The transition to parenthood represent a critical period for determining both mental and physical health in midlife and beyond [20].

The aim of the present study was to study Adverse Childhood Experiences (ACE) in couples and to examine if a person with an increased level of ACEs was more likely to have a partner with similar experiences. An additional aim was, to investigate the association between the relationship of the load of ACE exposure within couples and social circumstances, relational aspects, health risk factors and health problems, in the sensitive period of transition to parenthood one year after the birth of a common child.

## Materials and methods

### Study design

This is a cross-sectional study where the members of 818 couples individually responded to questionnaires, including questions about ACEs one year after the birth of a common child.

### Setting

The study is based on data from the first and last of three questionnaires (Q1, Q2, Q3) distributed in early pregnancy, in late pregnancy and one year postpartum, to the women who were registered in the national antenatal screening programme from 2012–2013. Exclusion criteria for Q3 were: reported miscarriage, multiple birth and not speaking Swedish. A corresponding questionnaire to the partners was distributed at the time for Q3.

One hundred and ninety-six antenatal clinics in Sweden were invited to participate, whereof 153 (71%) accepted the invitation. Antenatal clinics in both rural and urban areas of the middle of Sweden participated, including clinics in multicultural areas. Data collected about women on highest attained education, origin and height were collected from Q1 and on age, weight, sociodemographic, psychosocial characteristics and ACEs from Q3. The study variables were the same for the women and the partners.

### Participants

During the recruitment period, there were 5796 women enrolled, of which 3389 (3389/5796 = 62%) chose to participate. The midwives at the antenatal clinics distributed Q1. The study group sent Q2 and Q3 to the participants by post together with a prepaid return envelope. Women who did not respond within two weeks received a reminder. Those who answered Q1 (3389) received Q2. The women who answered Q2 received Q3 (2018/3389 = 60%) whereof 1257 women (1257/2018 = 62%) answered Q3. Among women answering Q3 who had a partner, 818 of the partners returned their questionnaire, one year after the birth of a common child (818/1257 = 65%). Thus, the study population comprised 818 couples: 14% of enrolments (818/5796), 24% of respondents to Q1 (818/3389), 40% of respondents to Q2 (818/2018) and 65% of respondents to Q3 (818/1257). Women responded to Q1 at a mean of 9.6 weeks of gestation (s.d. 3.5), Q2 at a mean of 31.9 (s.d. 1.7) weeks of gestation and Q3 at a mean of 52.8 (s.d. 3.9) weeks postpartum. From now on we use the word mother instead of woman.

### Variables

When no established cut-off value to a particular variable was available we chose a cut-off to represent the <25^th^ percentile or >75^th^ with the worst outcome of this particular variable.

#### Exposure variable

The survey of ACEs originates from a study conducted by Felitti et al. 1998, encompassing 10 ACE categories, including questions on neglect that were added during the study's second wave [6]. To create a Swedish version, the questions were translated from English into Swedish and then translated back into English. This tested their accuracy by comparison with the original questionnaire.

All ACE questions referred to the participants’ (the mothers and their partners) first 18 years of life. Each question was answered either by a five-level scale (never/once or twice/sometimes/often/very often) or by the dichotomous no/yes. Between one and eight questions were asked for each ACE category, coded as experienced or not experienced [6]. The number of experienced ACE categories was added up to produce an ACE score ranging from zero to 10.

To investigate the effect of ACE category exposure within couples, the mothers and their partners were assigned to one of five groups of couples: mothers no ACE category/partners no ACE category, both members 0–3 categories, no ACE category for either partner, mothers ≥4 categories and partners 0–3 categories, mothers 0–3 categories and partners ≥4 categories and both members ≥4 categories. The limit of ≥4 ACE categories was chosen according to the comprehensive study by Hughes [4].

Each ACE category was coded as not experienced until any of the criteria described below was true:

*Emotional abuse* was coded as experienced if the answer was often or very often to at least one of the questions ‘How often did it occur that a parent, stepparent, or adult living in your home swore at you, insulted you, or put you down? or acted in a way that made you afraid that you might be physically hurt?’.

*Physical abuse* was coded as experienced if the answer was any other than never, to at least one of the two questions ‘How often did it occur that a parent, stepparent, or adult living in your home actually pushed, grabbed, shoved, slapped, or threw something at you? or ‘Hit you so hard that you had marks or were injured?’. This was an alternation from the cut-off chosen by Felitti et al. [6] as all kind of child corporal punishment is prohibited by Swedish law.

*Sexual abuse* consisted of four questions asking whether an adult, older relative, family friend or stranger at least five years older than themselves had ever ‘Touched or fondled your body in a sexual way?’ or ‘Had you touch or fondle your body in a sexual way?’ or ‘Attempted to have any type of sexual intercourse (oral, anal, or vaginal) with you?’ or ‘Actually had sexual intercourse (oral, anal, or vaginal) with you?’. If it had happened prior to the age of 15 the ACE was coded as experienced if the answer was ‘yes’ to any of the four questions, irrespective of if it was of one’s own free will. If it had happened from the age of 15 until the 18^th^ birthday it was only coded as experienced if it had happened against one’s own free will. This alternation on age was made in accordance with Swedish legislation on sexual offence where sexual contact on one’s own free will from the age of 15 is not criminalized.

*Emotional neglect* consisted of the two questions ‘How often did you feel that no one in your family loved you or thought you were important or special? and in your family people did not look out for each other, feel close to each other or support each other?’ (merged together from five questions in the original). One point was given for each step in the five-level scale, thus the total score for the two questions ranged from two to 10. This ACE category was coded as experienced if the total score was six or more. In the original, the five questions were coded as experienced if the total score was 15 or more, thus the same proportions were kept.

*Physical neglect* consisted of the two questions ‘How often did you not have enough to eat, had to wear dirty clothes, or did not know there was someone to take care of you?’ and ‘How often were your parents too drunk or high to take care of you or take you to the doctor if you needed it?’ (merged together from five questions in the original). This ACE category was coded as experienced in the same way as emotional neglect but with a total score of four or more considered as experienced.

*Domestic violence* consisted of questions asking how often a father, stepfather or mother’s boyfriend/partner did anything of the following towards the mother or stepmother: ‘Pushed, grabbed, slapped or threw something at her?’, ‘Kicked, bit or hit her with a fist or something hard?’, ‘Repeatedly hit her over at least a few minutes?’ or ‘Threatened her with a knife or firearm, or used a knife or firearm to hurt her?’. The same questions were asked whether a mother, stepmother or father’s girlfriend/partner had done any of the four listed actions towards the father or stepfather. This ACE category was coded as experienced if the answer was sometimes, often or very often to any of the first two questions, or any other answer than never to the third or fourth question, either the mother or the father being the victim.

*Domestic substance abuse* was coded as experienced if the answer was that the participant had lived together with a problem drinker, alcoholic or someone using [illegal] drugs.

*Domestic mental illness* was coded as experienced if the answer was that someone in the participant’s home had been depressed, mentally ill or had attempted to commit suicide.

*Incarcerated domestic member* was coded as experienced if the answer was that someone in the participant’s home had ever gone to prison.

*Parental separation or divorce* was coded as experienced if the answer was that the participant’s parents were ever separated or divorced.

#### Outcome variables

*Social circumstances*. Highest attained education was reported on a 7 point Likert scale between no education and university studies with doctoral degree, dichotomized between points ≤3 (“Low level of education” equals to high school) and ≥4 (qualified professional training or higher) corresponding to the 34^rd^ percentile for the mothers and 46^th^ percentile for the partner.

Monthly household income was presented on an 11 point Likert scale where each point indicates 10^4^ SEK between 0 SEK and ≥10^5^ SEK as the total monthly household income before taxes (corresponding to intervals of approximately USD 930). The variable was dichotomized between points ≤2 (≤29,999 SEK) (“Low monthly income”) and ≥3 (≥30,000 SEK) corresponding to the 17^th^ percentile for the mothers and 15^th^ percentile for the partners.

*Relational aspects*. Feeling alone, was assessed by the study-specific question *‘Have you felt alone and isolated since you had a child*?*’* and answered on a 5-point Likert scale with alternatives from *‘Very often ‘* to *‘Very seldom’*. “Feeling alone” was defined as a score of ≤2, corresponding to the 20^th^ percentile for the mothers and 12^th^ percentile for the partners.

Cooperation and support from the spouse was assessed by the answers to the study-specific question *‘How satisfied are you with the cooperation and support from your partner after the birth of the child*?*’* in the three dimensions *‘Cooperation’*, *‘Emotional support’* and *‘Practical support’* reported on three 5-point Likert scales giving sum scores of 3 to 15. “Bad spousal support” was defined as a sum score of ≥7 points, corresponding to 80^th^ percentile for mothers and partners.

Relationship Assessment Scale (RAS) was used to measure general relationship satisfaction. RAS is a 7-item, 5-point Likert scale., with a total sum score ranging from 7 to 35, where a higher sum score the more satisfied respondent. The individual mean of the total sum score was calculated [21]. The higher the mean score the more satisfied respondent. “Low RAS score” was defined as a mean value <3.72, corresponding to 25^th^ percentile for mothers and partners.

*Health risk factors*. Cigarette smoking was coded if the answers to the question “*Do you smoke at present*?” were *‘Yes*, *I smoke every day’*, or *‘Yes*, *but not every day’*.

Weekly alcohol use was coded if the answer to the question *‘Do you drink alcohol at present*?*’* was *‘Yes*, *every week’*.

The body-mass index (BMI) is the weight in kilograms divided by the square of the height in meters. Obesity was defined as BMI≥30.0 kg/m^2^.

Sense of coherence, was assessed by a 3-item SOC questionnaire (SOC-3) [22]. The questionnaire assessed each of the component constructs (comprehensibility, manageability and meaningfulness) by the questions: *Do you usually feel that the things that happen to you in your daily life are hard to understand*?, *Do you usually see a solution to problems and difficulties that other people find hopeless*? and *Do you usually feel that your daily life is a source of personal satisfaction*?. The answers were ‘Yes, most often’, ‘Yes, sometimes’ or ‘No’ with the most positive answers giving 3 points, and the least positive giving 1 point. All items were summed to provide a total SOC-3 scale score in the range of 3 to 9. “Low SOC-3 score” is defined as a sum <7 points [22].

*Health problems*. Tired and out of form, was assessed by a study specific question *‘Because of changed sleep pattern I often feel tired and out of form’* and answered on a 5-point Likert scale from the alternative *‘Does not apply at all’* to *‘Applies very well’*. “Tired and out of form” was defined as a score of ≥4, corresponding to the 54^th^ percentile for the mothers and 59^th^ percentile for the partners.

Self-rated general health was assessed by the question *‘How do you judge your general state of health at present*?*’* with the answers on a 5-point Likert scale where 1 means *‘Very good’* and 5 means *Very Bad’*. “Bad assessed health” is defined as a score of ≥3 [23].

Perceived Stress Scale 10 (PSS-10), a 10-item, 5-point Likert scale giving sum scores from 0 to 40, was used to assess perceived stress during the last month. The PSS-10 has been psychometrically evaluated and is suggested as a global scale for this purpose [24]. “High perceived stress” is defined as sum of ≥19 points [24].

Hospital Anxiety and Depression Scale (HADS), the anxiety part, a 7-item, 4-point Likert scale giving sum scores of 0 to 21, was used to assess anxiety [25]. The participants had to assess their feelings during the past week. “Probable anxiety” is defined as a sum score of ≥11 [25].

Edinburgh Postnatal Depression Scale, EPDS, a 10 item, 4-point Likert scale validated in Norway [26], giving sum scores of 0 to 30, was used to assess depression [27]. “Probable depression” is defined as a sum score ≥13 [27].

#### Covariate

Non-Swedish origin was coded if the study person, his or her parents were born outside Sweden.

### Ethical approval

The study was approved by the Regional Ethics Board in Uppsala, Sweden (Dnr 2010/085). Written informed consent was obtained.

### Statistical methods

Summary statistics were calculated using standard methods, with frequencies for the categorical variables, median and interquartile range (IQR) for ordinal and skewed data and mean and standard deviation for continuous variables. To test the probability of no difference between mothers and partners χ^2^-test or Wilcoxon’s non-parametric test was used.

The Spearman's rank correlation coefficient was used to calculate the strength and direction of the relationship. Robust Linear regression analysis was used to calculate the parameter estimate of the association between the score of ACEs of mothers versus that of their partners.

To analyse the odds that a partner reported an ACE category (dependent variable) given the corresponding mother had or had not experienced the same ACE category (independent variable) we used logistic regression analyses reported as crude and adjusted odds ratios (OR) and the corresponding 95% confidence intervals (CI). The potential confounding factors adjusted for were non-Swedish origin and highest attained education.

To test associations between groups of couples regarding ACE category exposure and dichotomized demographic and psychosocial characteristic variables we used Mantel-Haenszel χ^2^-test. A p-value of <0.05 was considered significant.

To analyse the odds of several outcomes among mothers, partners, and couples (both members) in relation to the groups of couples regarding ACE category exposure (see paragraph above) we used ordinal logistic regression analyses reported as adjusted OR and the corresponding 95% CI. The couples with none of the members reporting ACE were used as reference.

Missing data were in general few. For the outcome measures, the proportions of missing data for mothers and partners varied between 0.2%-3.4%, except for age of mothers where 5.3% were missing. For the exposure variable ACE categories, the absolute number of participants with complete data are given in Tables 2 and 3. Across each separate ACE category missing values varied between 0.1%-0.6% in mothers and 1.3%-2.3% in partners. At least one missing ACE category was shown in 21 mothers (2.6%) and in 43 partners (5.3%) of the partners. At least one missing value for an ACE category occurred in 2 couples (0.24%). The sum of missing values of ACE categories among women and partners showed no correlation (Spearman correlation coefficient ρ = 0.06, p = 0.08). The sum of missing values of ACE categories within the couples showed no correlation to the outcome variables, except income (ρ = -0.07, p = 0.03) and self-rated health (ρ = -0.08, p = 0.02) among the partners and to BMI (ρ = -0.08, p = 0.03) and RAS score (ρ = 0.07, p = 0.04) among the mothers.

In an analysis of non-response, the results in Q1 of the included mothers whose partners responded to the Q3 (n = 818) were compared to the results in Q1 of those women who were not included in the study since she or her partner did not respond to the questionnaire. In the analysis the variables age, BMI, origin, highest attained education, cigarette smoking, weekly alcohol and number of previous deliveries reported in Q1 were compared by Wilcoxon’s non-parametric test. The mothers included in the present study (n = 818), compared to women who only answered the Q1 (n = 2571) showed higher proportions of: ≥3 years university studies (55% vs 38.9%), at least part time employment (88.2% vs 81.2%) and Swedish origin (82.3% vs 74.7%) while BMI and age showed no statistical difference.

Statistical analyses were performed using the SAS program, version 9.4 (SAS Institute Inc., Cary, NC, USA).

## Results

Table 1 shows the characteristics of the 818 mothers and their partners one year after the birth of a common child. One year postpartum, all mothers but one had the same partner as in early pregnancy.

**Table 1:** Characteristics of mothers and partners within 818 couples one year after the birth of a common child. Number (%), mean (standard deviation (s.d.)) and median (interquartile range (IQR)) are presented.

Characteristic	n = ^1)^	Mothers^2)^	n = ^1)^	Partners^2)^	p^3)^
Age (yr)–mean (sd)	775	31.3 (4.6)	804	34.3 (5.6)	<0.001
Non-Swedish origin—numbers (%)	807	144 (17.8)	800	132 (16.5)	0.508
Highest attained education^4)^ - median (IQR)	805	6 (3)	815	4 (3)	<0.001
Monthly household income (SEK)^5)^ –median (IQR)	790	4 (2)	816	4 (2)	0.006
Feeling alone^6)^ –median (IQR)	803	4 (2)	809	4 (2)	0.057
Cooperation and support from spouse^7)^ –median (IQR)	803	4 (3)	806	4 (3)	0.690
RAS mean score^8)^ –median (IQR)	803	4.4 (0.9)	808	4.3 (0.9)	0.030
Cigarette smoking^9)^ - numbers (%)	809	34 (4.2)	808	61 (7.6)	0.004
Weekly alcohol use—numbers (%)	809	127 (15.7)	809	279 (34.5)	<0.001
BMI^10)^ (kg/m^2^)–mean (sd)	791	24.8 (4.8)	813	25.9 (3.6)	<0.001
SOC-3 score^11)^ –median (IQR)	808	8 (1)	799	8 (1)	0.136
Tired and out of form^12)^ –median (IQR)	807	3 (2)	807	3 (2)	0.038
Self-rated health^13)^ –median (IQR)	808	2 (0)	806	2 (1)	<0.001
PSS-10 score^14)^ –median (IQR)	809	14 (9)	805	14 (9)	0.254
HADS score, anxiety part^15)^ –median (IQR)	809	4 (5)	802	3 (4)	0.034
EPDS score^16)^ –median (IQR)	809	4 (6)	808	4 (5)	<0.001
Previous deliveries—numbers (%)	810	420 (51.8)	-	-	-
Male sex of partner—numbers (%)	-	-	815	800 (98.2)	-
Cohabiting with the partner in early pregnancy—numbers (%)	802	791 (98.6)			

^1)^ Effective responses, n.

^2)^ Answers, n (%).

^3)^ The probability of no difference between the groups was tested by χ^2^-test, Mantel-Haenszel χ^2^-test or Wilcoxon’s non-parametric test.

^4)^ Highest attained education, was reported on a 7 point Likert scale between no education and university studies with doctoral degree.

^5)^ Monthly household income, presented on an 11 point Likert scale where each point indicates 10000 SEK between 0 SEK to ≥10^5^ SEK/month. SEK = Swedish Krona, 1 SEK = 0.123 US dollar.

^6)^ Feeling alone, was reported on a 5-point Likert scale where 1 means “Very often” and 5 means “Very seldom”.

^7)^ Cooperation and support from spouse, was assessed in three dimensions: cooperation, psychologic and practical support, each on a 5-point Likert scale where 1 means “very satisfied” and 5 means “very dissatisfied”, giving a total sum between 3 and 15.

^8)^ Relationship assessment scale (RAS). RAS consists of 7 items, 5-point Likert scale with a total sum score ranging from 7 to 35, where a higher sum score means higher satisfaction with the relationship. The individual mean of the total sum score was calculated.

^9)^ Cigarette smoking: “daily smoking” or “smoking but not every day”.

^10)^ BMI = body mass index (kg/m^2^).

^11)^ Sense of coherence-3 (SOC-3), a 3 items, 3-point Likert scale with the sum ranging from 3 to 9, where a higher sum means higher sense of coherence.

^12)^ Tired and out of form because of changed sleep pattern. A study specific question on a 5-point Likert scale, with the alternatives “do not agree” to “totally agree”.

^13)^ Self-rated health, was assessed on a 5 point Likert scale where 1 means “very good” and 5 means “very bad”.

^14)^ Perceived stress scale-10 (PSS-10), a 10 items, 5-point Likert scale with the sum ranging from 10 to 50, where a higher sum means higher perceived stress.

^15)^ Hospital Anxiety and Depression Scale (HADS). The anxiety part held 7-items, 4-point Likert scale with a sum-score range 0–21, where a higher sum means more anxiety symptoms.

^16)^ Edinburgh Postnatal Depression Scale (EPDS), 10 items, 4-point Likert scale giving sum scores of 0–30, where a higher sum means more depressive symptoms.

Table 2 shows the frequencies of ACE categories reported by the mothers, the partners and couples (reported by both members). Parental separation or divorce was the most commonly reported ACE category and physical abuse was the second most commonly reported ACE category. There was no difference of the different ACE categories between mothers and partners, except for the categories sexual abuse, emotional neglect and domestic mental illness which were more commonly reported by mothers (<0.001<p<0.024).

**Table 2:** Exposure to Adverse Childhood Experience (ACE) categories reported one year after the birth of a common child among 818 couples where both members reported the ACE experience and separate figures for the mothers and the partners. Number and proportions (%) are presented.

ACE categories	Both members in the couples		Mothers		Partners		
	n^1)^	n (%)^2)^	n^1)^	n (%)^2)^	n^1)^	n (%)^2)^	p^3)^
Sexual abuse	791	8 (1.0)	807	70 (8.7)	800	12 (1.5)	<0.001
Emotional abuse	795	6 (0.8)	807	39 (4.8)	805	29 (3.6)	0.220
Incarcerated domestic member	788	1 (0.1)	806	13 (1.6)	799	19 (2.4)	0.273
Domestic violence	788	9 (1.1)	805	57 (7.1)	799	52 (6.5)	0.649
Domestic substance abuse	793	40 (5.0)	809	137 (16.9)	801	144 (18.0)	0.582
Physical abuse	798	71 (8.9)	808	184 (22.8)	807	214 (26.5)	0.081
Physical neglect	795	5 (0.6)	807	42 (5.2)	803	52 (6.5)	0.277
Parental separation or divorce	792	89 (11.2)	808	221 (27.4)	801	252 (31.5)	0.071
Domestic mental illness	789	25 (3.2)	807	147 (18.2)	799	103 (12.9)	0.003
Emotional neglect	795	35 (4.4)	807	167 (20.7)	803	131 (16.3)	0.024
**Accumulated ACE categories**							
No ACE category	817	156 (19.2)	811	335 (41.4)	810	335 (41.4)	0.672
ACE categories ≥4	803	15 (1.9)	810	89 (11.0)	810	71 (8.8)	0.134

^1)^ Effective responses, n.

^2)^ Answers, n (%).

^3)^ The probability of no difference between the mothers and their partners was analyzed with χ^2^-test.

Table 2 also shows, frequencies of accumulated ACE categories reported by the mothers, the partners and couples (reported by both members). Within couples where both members had the same load of ACE experience no ACE category at all was found in 156 (19.2%) couples, 1–3 ACE categories in 190 (23.3%) couples and ≥4 ACE categories in 15 (1.9%) couples. Furthermore, in total 59% of both mothers and partners reported exposure to at least one any of the ten ACE categories.

**Fig 1:**
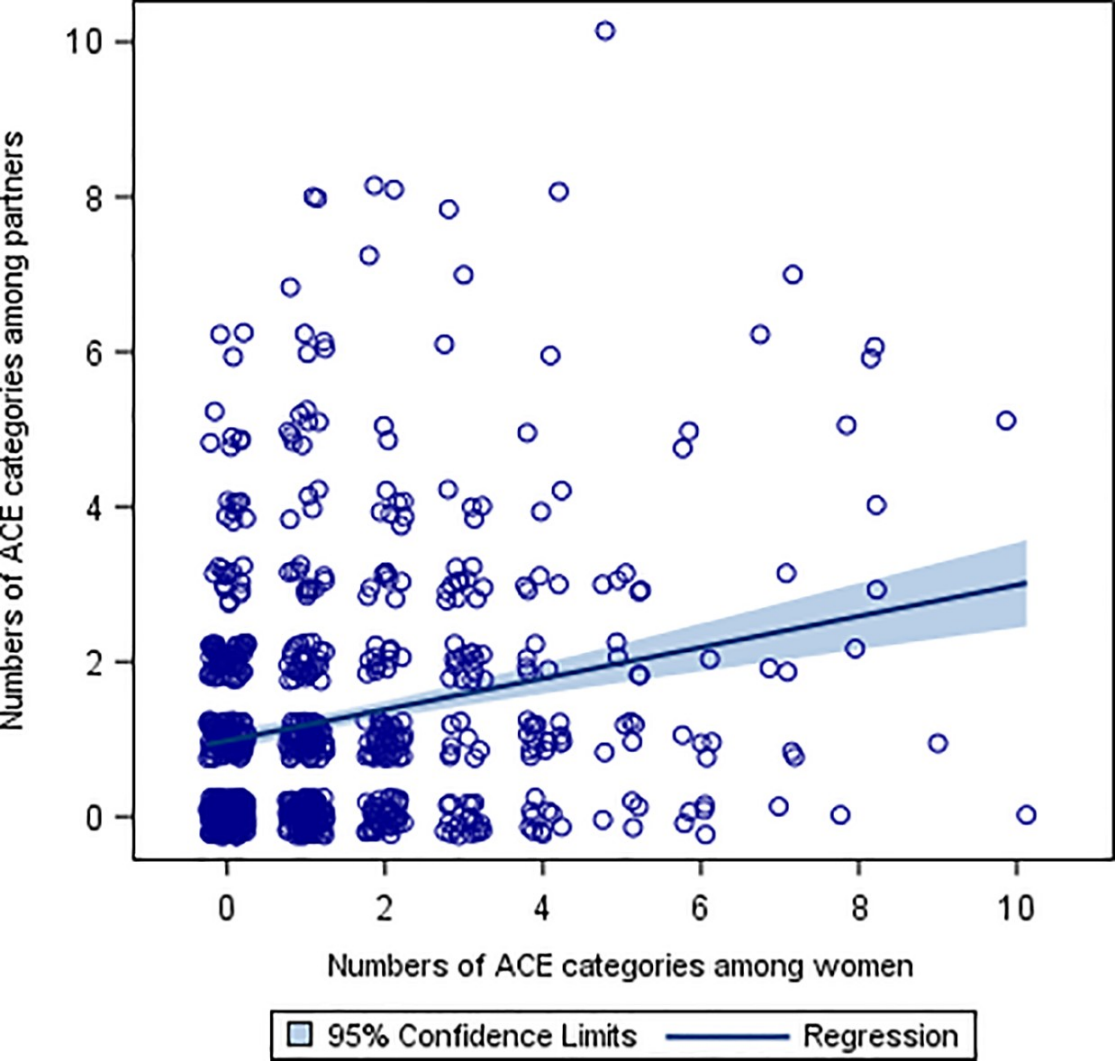
Numbers of Adverse Childhood Experience (ACE) categories reported by the mothers and their partners.

Fig 1 shows the numbers of ACE categories reported by the mothers and their partners. There was a positive correlation between the numbers of ACE categories among the mothers and the partners (Spearman’s ρ = 0.18, p<0.001). In a corresponding robust regression analysis the unadjusted parameter estimate (β) was 0.16 (p<0.0001). This means that a change of 5 units of ACE score of the mothers increases the partners’ ACE score with almost one unit. After adjustment for potential confounding factors (non-Swedish origin and level of education) the ACE score of the mother was independently associated to the ACE score of the partners (β = 0.15, p<0.0001).

Table 3 shows the OR (CI) that a partner reported an ACE category given the corresponding mother had or had not experienced the same ACE category. When a mother had experienced a certain ACE category the OR for her partner to have the same experience was between 1.5 and 23.1. The highest ORs were displayed for sexual abuse, emotional abuse and incarcerated domestic member. The ORs remained about the same after adjusting for origin and level of education.

**Table 3:** Odds ratios of Adverse Childhood Experience (ACE) categories reported by the partner, one year after the birth of a common child, given that the mother had the same experience, among 818 couples. Crude odds ratios (cOR) with 95% confidence intervals (CI) and odds ratios adjusted by origin and level of education (aOR) with 95% CI.

ACE categories	n = ^1)^	cOR	CI	p	n = ^1)^	aOR	CI	p
Sexual abuse (no/yes)	791	23.13	6.77–78.97	<0.001	752	15.16	3.80–60.50	<0.001
Emotional abuse (no/yes)	795	5.80	2.21–15.19	<0.001	756	4.12	1.47–11.51	0.007
Incarcerated domestic member (no/yes)	788	3.50	0.43–28.42	0.240	747	3.18	0.36–28.11	0.299
Domestic violence (no/yes)	788	3.00	1.38–6.52	0.006	752	2.46	1.08–5.60	0.032
Domestic substance abuse (no/yes)	793	2.42	1.47–3.43	<0.001	752	2.12	1.34–3.35	0.001
Physical abuse (no/yes)	798	2.13	1.50–3.03	<0.001	759	2.02	1.40–2.94	<0.001
Physical neglect (no/yes)	795	2.13	0.80–5.67	0.132	755	2.19	0.76–6.32	0.146
Parental separation or divorce (no/yes)	792	1.81	1.31–2.51	<0.001	752	1.67	1.18–2.36	0.004
Domestic mental illness (no/yes)	789	1.57	0.96–2.56	0.075	748	1.47	0.88–2.45	0.145
Emotional neglect (no/yes)	795	1.47	0.96–2.26	0.080	755	1.50	0.95–2.35	0.080
**Accumulated ACE categories**								
No ACE category	803	1.49	1.12–1.98	0.007	762	1.43	1.06–1.93	0.001
ACE categories ≥4	803	2.43	1.31–4.51	0.005	762	2.24	1.16–4.30	<0.001

^1)^ n = number of couples with complete data.

Table 4 shows proportions of dichotomized outcome measures in mothers, partners, and couples with increased exposure of ACE categories from 0 to ≥4. In general, there was a positive trend to an unfavourable outcome with higher exposure of ACE categories among the mothers, partners and within the couples.

**Table 4:** Proportions of outcome measures related to groups of couples with different numbers of ACE category exposure: 0) both members no category (n = 156), A) both members 0–3 categories, except both members no category (n = 503), B) mothers ≥4 categories and partners 0–3 categories (n = 74), C) mothers 0–3 categories and partners ≥4 categories (n = 55) and D) both members ≥4 categories (n = 15). Numbers and proportions (%) are presented.

	Groups of ACE category exposure					
**Mothers’ outcomes**	0	A	B	C	D	p^2)^
*Background variables*						
Low level of education^3)^	42/155 (27.1)	156/497 (31.4)	38/71 (53.5)	21/53 (39.6)	11/15 (73.3)	<0.001
Low monthly income^4)^	14/152 (9.2)	79/488 (16.2)	18/74 (24.3)	16/55 (29.1)	8/15 (53.3)	<0.001
*Relational aspects*						
Feeling alone^5)^	15/155 (9.7)	99/498 (19.9)	22/73 (30.1)	10/55 (18.2)	7/15 (46.7)	<0.001
Bad spousal support^6)^	29/156 (18.6)	94/497(18.9)	17/73 (23.3)	11/55 (20.0)	8/15 (53.3)	0.041
Low RAS score^7)^	29/156 (18.6)	96/497 (19.3)	20/73 (27.4)	13/55 (23.6)	9/15 (60.0)	0.003
*Health risk factors*						
Cigarette smoking^8)^	3/155 (1.9)	21/503 (4.2)	4/74 (5.4)	2/55 (3.6)	3/15 (20.0)	0.022
Weekly alcohol use	24/155 (15.5)	83/503 (16.5)	11/74 (14.9)	8/55 (14.6)	1/15 (6.7)	0.515
Obesity ^9)^	12/153 (7.8)	63/489 (12.9)	14/74 (18.9)	12/55 (21.8)	4/14 (28.6)	<0.001
Low SOC-3 score^10)^	20/154 (13.0)	83/503 (16.5)	15/74 (20.3)	15/55 (27.3)	8/15 (53.3)	<0.001
*Health problems*						
Tired and out of form^11)^	72/154 (46.8)	219/503 (43.5)	40/74 (54.0)	27/55 (49.1)	9/15 (60.0)	0.221
Bad assessed health^12)^	30/155 (19.4)	110/502 (21.9)	20/74 (27.0)	16/55 (29.1)	8/15 (53.3)	0.004
High PSS-10 score^13)^	35/156 (22.4)	119/503 (23.7)	29/74 (39.2)	21/55 (38.2)	10/15 (66.7)	<0.001
Probable anxiety^14)^	3/156 (1.9)	37/503 (7.4)	8/74 (10.8)	8/54 (14.8)	7/15 (46.7)	<0.001
Probable depression^15)^	3/155 (1.9)	42/503 (8.4)	11/74 (14.9)	8/55 (14.6)	5/15 (33.3)	<0.001
**Partners’ outcomes**	0	A	B	C	D	p^2)^
*Background variables*						
Low level of education^3)^	55/154 (35.7)	236/502 (47.0)	37/74 (50.0)	25/55 (45.4)	12/15 (80.0)	0.005
Low monthly income^4)^	13/154 (8.4)	69/503 (13.7)	17/74 (23.0)	9/55 (16.4)	5/15 (33.3)	0.002
*Relational aspects*						
Feeling alone^5)^	9/156 (5.8)	65/502 (13.0)	8/73 (11.0)	12/55 (21.8)	3/15 (20.0)	0.003
Bad spousal support^6)^	28/153 (18.3)	84/502 (16.7)	14/73 (19.2)	24/55 (43.6)	6/15 (40.0)	<0.001
Low RAS score ^7)^	31/155 (20.0)	109/502 (21.4)	21/73 (28.8)	20/55 (36.4)	4/15 (26.7)	0.013
*Health risk factors*						
Cigarette smoking^8)^	9/155 (5.8)	32/502 (6.4)	7/73 (9.6)	7/55 (12.7)	5/15 (33.3)	<0.001
Weekly alcohol use	64/156 (41.0)	168/502 (33.5)	20/73 (27.4)	23/55 (41.8)	1/15 (6.7)	0.083
Obesity^9)^	12/155 (7.7)	59/499 (11.8)	8/74 (10.8)	10/55 (18.2)	5/15 (33.3)	0.004
Low SOC-3 score^10)^	20/151 (13.2)	76/499 (15.2)	15/73 (20.6)	15/54 (27.8)	4/15 (26.7)	0.005
*Health problems*						
Tired and out of form^11)^	57/154 (37.0)	200/503 (39.8)	36/73 (49.3)	26/55 (47.3)	7/15 (46.7)	0.064
Bad assessed health^12)^	34/155 (21.9)	168/501 (33.5)	29/74 (39.2)	30/54 (55.6)	10/15 (66.7)	<0.001
High PSS-10 score ^13)^	27/154 (17.5)	123/501 (24.6)	23/74 (31.1)	20/54 (37.0)	9/15 (60.0)	<0.001
Probable anxiety^14)^	3/152 (2.0)	22/501 (4.4)	7/73 (9.6)	5/54 (9.3)	4/15 (26.7)	<0.001
Probable depression^15)^	4/156 (2.6)	27/502 (5.4)	5/74 (6.8)	7/54 (13.0)	3/15 (20.0)	<0.001
**Couples’ outcomes**	0	A	B	C	D	p^2)^
*Background variables*						
Low level of education^3)^	29/153 (19.0)	122/500 (24.4)	20/71 (28.2)	11/54 (20.4)	10/15 (66.7)	0.012
Low monthly income^4)^	10/155 (6.4)	53/501 (10.6)	12/74 (16.2)	9/55 (16.4)	5/15 (33.3)	<0.001
*Relational aspects*						
Feeling alone^5)^	1/156 (0.6)	23/502 (4.6)	3/74 (4.0)	3/55 (5.4)	1/15 (6.7)	0.078
Bad spousal support^6)^	8/156 (5.1)	28/500 (5.6)	7/74 (9.5)	5/55 (9.1)	5/15 (33.3)	0.001
Low RAS score^7)^	15/156 (9.6)	49/500 (9.8)	12/73 (16.4)	10/55 (18.2)	4/15 (26.7)	0.006
*Health risk factors*						
Cigarette smoking^8)^	0/156 (0)	10/503 (2.0)	3/74 (4.0)	0/55 (0)	3/15 (20.0)	0.002
Weekly alcohol use	22/155 (14.2)	55/502 (11.0)	5/74 (6.8)	7/55 (12.7)	0/15 (0)	0.137
Obesity^9)^	5/155 (3.2)	15/501 (3.0)	5/74 (6.8)	4/55 (7.3)	3/14 (21.4)	0.002
Low SOC-3 score^10)^	5/154 (3.2)	18/501 (3.6)	4/74 (5.4)	6/55 (10.9)	3/15 (20.0)	<0.001
*Health problems*						
Tired and out of form^11)^	32/154 (20.8)	110/503 (21.9)	19/73 (26.0)	13/55 (23.6)	6/15 (40.0)	0.152
Bad assessed health^12)^	8/155 (5.2)	54/501 (10.8)	11/74 (14.9)	12/55 (21.8)	6/15 (40.0)	<0.001
High PSS-10 score^13)^	9/156 (5.8)	40/502 (8.0)	11/74 (20.4)	11/54 (20.4)	9/15 (60.0)	<0.001
Probable anxiety^14)^	0/156 (0)	3/502 (0.6)	2/73 (2.7)	3/55 (5.4)	3/15 (20.0)	<0.001
Probable depression^15)^	1/156 (0)	3/503 (0.6)	2/74 (2.7)	1/54 (1.8)	2/15 (13.3)	<0.001

^2)^ The probability of no difference of the trend across the couples regarding the exposure of ACE categories (Mantel-Haenszel χ^2^-test).

^3)^ Highest attained education was reported on a 7-point Likert scale between no education and university studies with doctoral degree. Low level of education was defined as ≤3 (34^th^ percentile for the women and 46^th^ for the partners).

^4)^ Low monthly household income<30000 SEK/month, presented on an 11 point Likert scale where each point indicates 10^4^ SEK between 0 SEK and ≥10^5^ SEK/month, dichotomized between points 2 and 3 (17^th^ percentile for the women and 15^th^ for the partners). SEK = Swedish Krona, 1 SEK = 0.123 US dollar.

^5)^ Feeling alone, was reported on a 5-point Likert scale where 1 means “Very often” and 5 means “Very seldom”. “Feeling alone” was defined as ≤2 (20^th^ percentile for the women and 12^th^ for the partners).

^6)^ Cooperation and support from spouse, was assessed in three dimensions: cooperation, psychologic and practical support, each on a 5-point Likert scale where 1 means “very satisfied” and 5 means “very dissatisfied”, giving a total sum between 3 and 15. “Bad spousal support” was defined as a sum score ≥7 (≥80^th^ percentile).

^7)^ Relationship assessment scale (RAS), on the relation at present, consisting of 7 items, 5-point Likert scales with a total sum score ranging from 7 to 35, where a higher sum means more satisfaction with the relationship. The individual mean of the total sum score was calculated. “Low RAS score” was defined as mean sum score ≤25^th^ percentile.

^8)^ Cigarette smoking: “daily smoking” and “smokes but not every day”.

^9)^ Obesity was defined as BMI≥30 kg/m^2^. BMI = body mass index (kg/m^2^).

^10)^ Sense of coherence-3 (SOC-3), a 3 items, 3-point Likert scale with the sum ranging from 3 to 9, where a higher sum means lower sense of coherence. “Low SOC-3 score” was defined as a sum ≥7.

^11)^ “Tired and out of form” because of changed sleep pattern. A study specific question on a 5-point Likert scale, with the alternatives “do not agree” to “totally agree”, dichotomized between point 3 and 4 (54^th^ percentile for the women and 59^th^ percentile for the partners).

^12)^ Self-rated health, was assessed on a 5 point Likert scale where 1 means “very good” and 5 means “very bad”. “Bad assessed health” was defined as a score of ≥3.

^13)^ Perceived stress scale-10 (PSS-10), a 10 items, 5-point Likert scales with the sum ranging from 10 to 50, where a higher sum means higher perceived stress. “High PSS-10 score” was defined as a sum ≥19.

^14)^ Hospital Anxiety and Depression Scale. The anxiety part held 7-items, 4-point Likert scales with a sum-score range 0–21, where a higher sum means more anxiety symptoms. “Probable anxiety” was defined as a sum score of ≥11.

^15)^ Edinburgh Postnatal Depression Scale, 10 items, 4-point Likert scale giving sum scores of 0–30, where a higher sum means more depressive symptoms. “Probable depression” was defined as a sum score ≥13.

Table 5 shows the results of multiple ordinal regression analyses of the outcomes among mothers, partners, and couples with different load of ACE category exposure with the group of couples where none in reported exposure to ACE as a reference, adjusted for the potential confounding factors origin and highest attained education among the mothers and the partners. Overall, there were associations between higher exposure of ACE categories and unfavourable outcomes, particularly for health problems. The unfavourable outcomes were most prominent in couples where both members reported exposure to ≥4 ACE categories. Among couples where one of the members reported exposure to ≥4 ACE categories there was a tendency that the most exposed member reported worse outcomes.

**Table 5:** Results of multiple ordinal regression calculations among couples with different numbers of ACE category exposure: A) both members 0–3 categories, except both members no category (n = 503), mothers ≥4 categories and partners 0–3 categories (n = 74), C) mothers 0–3 categories and partners ≥4 categories (n = 55) and D) both members ≥4 categories (n = 15), with the group where none in the couple reported exposure to ACE (n = 156) as reference. The associations were adjusted by origin and level of education of the mothers and the partners. The results are presented as adjusted odds ratios with 95% confidence intervals (CI).

	Groups of ACE category exposure				
**Mothers’ outcomes**	A	B	C	D	p^2)^
*Background variables*					
Low level of education^3)^	1.25 (0.83–1.87)	*3*.*14 (1*.*73–5*.*69)*	1.78 (0.92–3.43)	*7*.*48 (2*.*25–24*.*85)*	<0.001
Low monthly income^4)^	1.81 (0.98–3.35)	2.05 (0.91–4.62)	*3*.*35 (1*.*44–7*.*78)*	*6*.*62 (2*.*00–22*.*00)*	<0.001
*Relational aspects*					
Feeling alone^5)^	*2*.*18 (1*.*22–3*.*90)*	*3*.*40 (1*.*60–7*.*24)*	2.00 (0.83–4.79)	*6*.*61 (2*.*07–21*.*13)*	<0.001
Bad spousal support^6)^	1.04 (0.64–1.67)	1.34 (0.67–2.71)	1.13 (0.51–2.49)	*4*.*23 (1*.*39–12*.*87)*	0.009
Low RAS score ^7)^	1.03 (0.64–1.65)	1.61 (0.82–3.16	1.08 (0.49–2.37)	*5*.*31 (1*.*72–16*.*38)*	0.001
*Health risk factors*					
Cigarette smoking^8)^	1.92 (0.55–6.64)	1.90 (0.40–9.00)	1.51 (0.24–9.53)	*6*.*22 (1*.*08–35*.*91)*	<0.001
Weekly alcohol use	1.13 (0.68–1.88)	1.13 (0.50–2.55)	1.08 (0.45–2.59)	0.54 (0.07–4.41)	0.145
Obesity^9)^	1.63 (0.85–3.13)	2.28 (0.98–5.34)	*2*.*69 (1*.*09–6*.*64)*	3.10 (0.82–11.72)	<0.001
Low SOC-3 score^10)^	1.22 (0.71–2.08)	1.09 (0.50–2.40)	2.02 (0.92–4.45)	*4*.*72 (1*.*50–14*.*91)*	<0.001
*Health problems*					
Tired and out of form^11)^	0.85 (0.58–1.23)	1.37 (0.76–2.46)	1.10 (0.58–2.07)	1.90 (0.63–5.76)	0.006
Bad assessed health^12)^	1.13 (0.71–1.80)	1.40 (0.72–2.75)	1.50 (0.72–3.12)	*3*.*51 (1*.*16–10*.*67)*	<0.001
High PSS-10 score ^13)^	1.00 (0.65–1.54)	*2*.*21 (1*.*19–4*.*10)*	1.94 (0.99–3.81)	*5*.*76 (1*.*82–18*.*20)*	<0.001
Probable anxiety^14)^	*3*.*80 (1*.*15–12*.*54*)	*5*.*68 (1*.*44–22*.*35)*	*7*.*20 (1*.*78–29*.*12)*	*35*.*34 (7*.*51–166*.*18)*	<0.001
Probable depression^15)^	*4*.*33 (1*.*32–14*.*20)*	*7*.*27 (1*.*91–27*.*64)*	*8*.*20 (2*.*08–32*.*39)*	*20*.*11 (4*.*10–98*.*66)*	<0.001
**Partners’ outcomes**	A	B	C	D	p^2)^
*Background variables*					
Low level of education ^3)^	*1*.*60 (1*.*10–2*.*33)*	*1*.*81 (1*.*03–3*.*18)*	1.57 (0.83–2.98)	*7*.*48 (2*.*01–27*.*77)*	0.021
Low monthly income^4)^	1.56 (0.82–2.95)	*2*.*88 (1*.*28–6*.*45)*	1.89 (0.74–4.85)	*2*.*71 (1*.*67–4*.*40)*	<0.001
*Relational aspects*					
Feeling alone^5)^	*2*.*36 (1*.*14–4*.*89)*	2.05 (0.76–5.58)	*4*.*48 (1*.*73–11*.*61)*	*4*.*56 (1*.*06–19*.*59)*	0.082
Bad spousal support^6)^	0.90 (0.56–1.44)	1.03 (0.50–2.10)	*3*.*14 (1*.*58–6*.*26)*	2.69 (0.87–8.32)	0.001
Low RAS score ^7)^	1.12 (0.71–1.77)	1.61 (0.84–3.08)	*2*.*08 (1*.*03–4*.*19)*	1.32 (0.39–4.52)	0.204
*Health risk factors*					
Cigarette smoking^8)^	0.97 (0.45–2.12)	1.48 (0.52–4.21)	2.05 (0.71–6.00)	*4*.*33 (1*.*14–16*.*45)*	<0.001
Weekly alcohol use	0.74 (0.51–1.08)	0.57 (0.31–1.05)	1.02 (0.54–1.95)	*0*.*12 (0*.*02–0*.*96)*	0.029
Obesity^9)^	1.58 (0.80–3.12)	1.36 (0.52–3.58)	2.41 (0.93–6.29)	*4*.*16 (1*.*17–14*.*80)*	<0.001
Low SOC-3 score^10)^	1.07 (0.62–1.83)	1.53 (0.73–3.22)	*2*.*30 (1*.*05–5*.*02)*	1.74 (0.49–6.15)	0.008
*Health problems*					
Tired and out of form^11)^	1.15 (0.78–1.67)	1.69 (0.96–2.98)	1.55 (0.82–2.95)	1.55 (0.52–4.58)	0.096
Bad assessed health^12)^	*1*.*75 (1*.*14–2*.*68)*	*2*.*19 (1*.*20–4*.*01)*	*3*.*81 (1*.*94–7*.*46)*	*6*.*13 (1*.*94–19*.*38)*	<0.001
High PSS-10 score ^13)^	1.41 (0.88–2.26)	*1*.*97 (1*.*03–3*.*78)*	*2*.*58 (1*.*27–5*.*23)*	*5*.*51 (1*.*78–17*.*08)*	<0.001
Probable anxiety^14)^	1.94 (0.57–6.67)	*4*.*75 (1*.*18–19*.*08)*	*5*.*17 (1*.*18–22*.*65)*	*14*.*84 (2*.*82–78*.*02)*	0.001
Probable depression^15)^	2.07 (0.71–6.03)	2.50 (0.65–9.65)	*4*.*62 (1*.*24–17*.*21)*	*6*.*97 (1*.*35–36*.*08)*	0.036
**Couples’ outcomes**^16)^	A	B	C	D	p^2)^
*Background variables*					
Low level of education ^3)^	*1*.*65 (1*.*02–6*.*68)*	*3*.*30 (1*.*52–7*.*16)*	1.86 (0.76–4.55)	*16*.*13 (3*.*30–78*.*90)*	0.005
Low monthly income^4)^	1.70 (0.81–3.57)	1.89 (0.70–5.09)	2.75 (0.97–7.78)	3.25 (0.77–13.68)	<0.001
*Relational aspects*					
Feeling alone^5)^	7.39 (0.98–55.89)	7.86 (0.78–79.30)	*11*.*03 (1*.*10–110*.*09)*	18.97 (0.96–375.82)	0.519
Bad spousal support^6)^	1.13 (0.47–2.68)	2.04 (0.67–6.28)	2.79 (0.80–9.70)	*9*.*39 (2*.*12–41*.*72)*	0.015
Low RAS score ^7)^	1.12 (0.58–2.17)	2.14 (0.88–5.18)	1.85 (0.70–4.92)	*4*.*10 (0*.*98–17*.*19)*	0.053
*Health risk factors*					
Cigarette smoking^8)^	*^)^	*^)^	1.92 (0.47–7.81)**^)^	*8*.*52 (1*.*83–39*.74)**^)^	0.001**^)^
Weekly alcohol use	0.92 (0.46–1.86)	0.48 (0.15–1.56)	1.05 (0.37–2.96)	*^)^	0.042
Obesity^9)^	0.91 (0.32–2.62)	1.75 (0.47–6.52)	1.14 (0.21–6.34)	3.92 (0.74–20.65)	0.005
Low SOC-3 score^10)^	1.15 (0.40–3.32)	1.29 (0.31–5.39)	3.44 (0.88–13.83)	5.80 (1.00–33.72)	<0.001
*Health problems*					
Tired and out of form^11)^	0.96 (0.58–1.61)	2.20 (0.96–5.01)	1.45 (0.60–3.53)	2.57 (0.67–9.80)	0.002
Bad assessed health^12)^	2.01 (0.91–4.45)	*2*.*82 (1*.*01–7*.*86)*	*4*.*37 (1*.*46–13*.*08)*	*14*.*73 (2*.*97–73*.*17)*	<0.001
High PSS-10 score^13)^	1.36 (0.63–2.93)	*3*.*25 (1*.*20–8*.*81)*	*4*.*18 (1*.*50–11*.*65)*	*11*.*78 (3*.*10–44*.*72)*	<0.001
Probable anxiety^14)^	*^)^	*15*.*16 (2*.*92–78*.64)**^)^	*6*.*37 (1*.*04–39*.12)**^)^	*91*.*97 (13*.*38–632*.07)**^)^	<0.001**^)^
Probable depression^15)^	*^)^	3.84 (0.37–39.73)**^)^	3.92 (0.56–27.53)**^)^	*17*.*42 (2*.*14–141*.78)**^)^	0.007**^)^

^2)^ Testing global null hypothesis.

^3)^ Highest attained education was reported on a 7-point Likert scale between no education and university studies with doctoral degree. Low level of education was defined as ≤3 (34^th^ percentile for the women and 46^th^ for the partners).

^4)^ Low monthly household income<30000 SEK/month, presented on an 11 point Likert scale where each point indicates 10^4^ SEK between 0 SEK and ≥10^5^ SEK/month, dichotomized between points 2 and 3 (17^th^ percentile for the women and 15^th^ for the partners). SEK = Swedish Krona, 1 SEK = 0.123 US dollar.

^5)^ Feeling alone, was reported on a 5-point Likert scale where 1 means “Very often” and 5 means “Very seldom”. “Feeling alone” was defined as ≤2 (20^th^ percentile for the women and 12^th^ for the partners).

^6)^ Cooperation and support from spouse, was assessed in three dimensions: cooperation, psychologic and practical support, each on a 5-point Likert scale where 1 means “very satisfied” and 5 means “very dissatisfied”, giving a total sum between 3 and 15. “Bad spousal support” was defined as a sum score ≥7 (≥80^th^ percentile).

^7)^ Relationship assessment scale (RAS), consisting of 7 items, 5-point Likert scales with a total sum score ranging from 7 to 35, where a higher sum means more satisfaction with the relationship. The individual mean of the total sum score was calculated. “Low RAS score” was defined as mean sum score ≤25^th^ percentile.

^8)^ Cigarette smoking: “daily smoking” and “smokes but not every day”.

^9)^ Obesity was defined as BMI≥30 kg/m^2^. BMI = body mass index (kg/m^2^).

^10)^ Sense of coherence-3 (SOC-3), a 3 items, 3-point Likert scale with the sum ranging from 3 to 9, where a higher sum means lower sense of coherence. “Low SOC-3 score” was defined as a sum ≥7.

^11)^ “Tired and out of form” because of changed sleep pattern. A study specific question on a 5-point Likert scale, with the alternatives “do not agree” to “totally agree”, dichotomized between point 3 and 4 (54^th^ percentile for the women and 59^th^ percentile for the partners).

^12)^ Self-rated health, was assessed on a 5 point Likert scale where 1 means “very good” and 5 means “very bad”. “Bad assessed health” was defined as a score of ≥3.

^13)^ Perceived stress scale-10 (PSS-10), a 10 items, 5-point Likert scales with the sum ranging from 10 to 50, where a higher sum means higher perceived stress. “High PSS-10 score” was defined as a sum ≥19.

^14)^ Hospital Anxiety and Depression Scale. The anxiety part held 7-items, 4-point Likert scales with a sum-score range 0–21, where a higher sum means more anxiety symptoms. “Probable anxiety” was defined as a sum score of ≥11.

^15)^ Edinburgh Postnatal Depression Scale, 10 items, 4-point Likert scale giving sum scores of 0–30, where a higher sum means more depressive symptoms. “Probable depression” was defined as a sum score ≥13.

^16)^ Couples’ outcomes were defined as 0 if both members reported no outcome and 1 if both members reported the outcome.

*^)^ Odds ratio could not be calculated.

**^)^ Odds ratios with groups of ACE category exposure B, C and D.

When answering the questionnaire, 42 out of 806 (5.2%) mothers and 90 of 802 (11.2%) partners had received help from their spouse or by someone else and 151 of 804 (18.8%) mothers and 235 of 802 (29.3%) partners had another person present when filling-in the questionnaire. There was no difference of numbers of ACE categories between individuals receiving help compared to no help (mothers, p = 0.227; partners, p = 0.188) or having another person present or not present (mothers, p = 0.289; partners p = 0.127).

In the sensitivity analyses between the group with any missing data of ACEs and the group with complete ACE data, there were no differences in outcome measures, except for non-Swedish origin for partners (16% vs 30%, p = 0.018). In the analyses of non-response between mothers whose partners did not participate compared with the answers of those whose partners did participate, there were no differences, except that the mothers with no participating partners were 1 year older (p = 0.026) and had lower attained education (p = 0.009).

## Discussion

### Major findings

This study is, to the best of our knowledge, the first to look at ACEs in couples from the general population. An association was found of the occurrence of number of ACE categories, within the couples, supporting the idea that ACEs might be of importance for assortative mating. Further, there was a relation between the exposure of ACE categories in the couples and the outcomes of social circumstances, relational aspects, health risk factors and health factors, such as self-rated health, anxiety, depression and perceived stress. When both members of the couples were exposed to ≥4 ACE categories the outcomes were most unfavourable.

### Limitations of the study

There were several selection steps of women to the study sample. Also, there was a selection due to participation of partners. This meant that the sample of participating mothers and partners might be skewed, since they might comprise a more privileged group than those originally approached. The retrospective design of the reporting of the ACE categories may inherit a possible recall bias. Because of these limitations, the real prevalence of the exposure of ACEs may have been underestimated, e.g. the low number of couples where both members reported exposure to ≥4 ACE categories. This is also true for the outcomes, e.g. anxiety and depression. We believe that the limitations had no detrimental influence on the validity of the reported associations. However, an inference of the results to the general population has to be made with caution.

In this study, we used the instrument of childhood adversities formulated by Felitti et al. [6], since it covers issues related to the family situation and therefore is the most appropriate for the study aim. There are other adverse exposures in children’s lives such as bullying and other peer related trauma, loss of significant others, war/refugee experiences and natural disasters that are not included [4] and not assessed in the present study. This might entail an underreporting of ACEs and misclassification of participants to the ACE group, which might have attenuated the associations.

Potential confounding factors not measured in the present study are related to the socioeconomic situation for the study participants’ original family. Highest attained education and reported origin of the study participants and their parents were controlled for as available indicators of the socioeconomic situation in the original family. However, education may partly be a mediator implying that the adjusted OR underestimates the true association. Another potential confounder was that some mothers and their partners may have co-operated when answering the questions in the questionnaires.

The study was conceived to ask mothers and partners about ACEs after the birth of a common child and to study the connection between the number of ACE categories between the mothers and their partners and possible outcomes individually. Following the analyses of these associations, the authors raised hypotheses about associations between ACEs within couples and health outcomes.

### Strengths of the study

A strength of the study was that the situation for couples in an important period of their lives, the transition to parenthood is highlighted. Another strength was the relatively high number of couples who participated. In addition, the ACE questionnaire is well known and used globally. This is also true for most of the other questionnaires. Furthermore, most results of the outcome measures point in the same direction with reasonable significance levels.

### Comparison with other studies

The prevalence of none, at least one and ≥4 ACE categories among mothers and partners in our study was in accordance with international studies [4]. National registry studies in Sweden showed similar levels of adversities when single individuals were studied [9]. We have not found other studies presenting the prevalence of ACEs within couples.

Assortative mating theories propose that individuals select partners who are similar to them in different aspects [28]. Our data support that ACEs may be of importance for the assortative mating, also when adjusting for origin and level of education. The importance of individuals with ACEs tending to have a partner with similar experiences can be interpreted in different ways. In the first phase of a pair relationship it might be positive to have a partner with similar experiences as a way of getting support and understanding. In further stages as the transition to parenthood it might instead cause strains in the relationship and negative effects on parenting [29].

From the research on ACEs, it is known that these events tend to cluster within individuals [15]. The new contribution from this paper was that ACEs also might cluster in couples. On the individual level Anda et al. described a graded relationship between the number of ACE categories and health outcomes [15], confirmed in further studies [4, 30]. In the couples where both members have multiple ACEs this exposure has an even stronger negative impact on health outcomes, anxiety, depression and stress. In addition, the outcomes most strongly associated with multiple ACEs also represent health risks for the next generation [31].

### Implications

Almost two thirds of the original participants in the study chose to respond and thus shared the important information. Therefore, it seems to be quite feasible to ask about ACEs and mental health and expect co-operation from the majority. The time interval around childbirth might thus constitute a window of opportunity [32]. Questions that would normally be regarded as an intrusion of privacy and cause unease in health care staff might thus be accepted willingly and this might help to understand better what kind of assistance might be needed in order to improve the future of the couple and the child [33, 34].

ACEs have profound effects on the lives and health of the affected individuals. This was most true for couples where both members were exposed to multiple ACEs, as shown in the present study. Global action to prevent and diminish these consequences must be the obligation for every society and is in accordance with the sustainable goals of the UN [32, 35]. These preventive and therapeutic efforts demand development of a new concept of primary care/general practice/health care as a whole, social welfare services as well as staff in schools with broadened competences in meeting, understanding and helping people afflicted by ACEs and other demanding life conditions [36]. The situation during pregnancy, preparing the couple for the birth, and parenthood might offer possibilities for information about the parents-to-be that would normally be considered highly private or even irrelevant to the medical system. The same is true in the sensitive period of transition to parenthood after the birth. In the same vein, we have to see and acknowledge experiences of refugees and migrants that have fled from war and disasters to other parts of the world in previous decades [37, 38]. We agree with Anda et al. [39] that it is important “to continue efforts by policy makers and legislators to provide knowledge and resources for human service systems as part of the rapidly growing movement to provide trauma-informed care and promote accurate and compassionate public understanding of adverse childhood experiences as an endemic public health problem”. To identify and support the individuals with ACEs is a huge challenge for the staff of human service systems.

## Conclusions

Mothers exposed to ACEs tend to have partners also exposed to ACEs. Exposure to ACEs was associated with bad health and unfavourable life conditions within the couples, especially among couples where both members reported exposure to multiple ACEs. These results should stimulate clinical incentives to find, to support and to treat mothers and partners as well as couples afflicted by ACEs. The consequences for the children should be further studied as well as how these families should be treated in health care and society.
